# Sublethal Effects of Cameroon Field-Used Pesticides on Growth and Organ Health in *Archachatina marginata*

**DOI:** 10.1155/jt/6365547

**Published:** 2025-09-12

**Authors:** Annick Niquaise Enangue Njembele, Sylvie C. Ntyam Epse Ondo, Kingsley Agbor Etchu

**Affiliations:** ^1^Specialized Research Station for Marine Ecosystems, Institute of Agricultural Research for Development, Kribi, South Region, Cameroon; ^2^Multipurpose Agricultural Research Station of Bertoua, Institute of Agricultural Research for Development, Bertoua, East Region, Cameroon; ^3^Institute of Agricultural Research for Development, Nkolbisson Headquarter, Nkolbisson, Yaoundé, Center Region, Cameroon

**Keywords:** agrochemical residues, environmental contaminants, mollusk toxicology, sublethal exposure outcomes, terrestrial invertebrates

## Abstract

Cameroon rainforest region is not only an agricultural area with massive pesticide uses but also possesses factors that favor land snails' growth like *Archachatina marginata*. The present study aimed to assess the impact of sub-chronic exposure of commonly used pesticides in Cameroon, glyphosate, metalaxyl, and cypermethrin on the growth, survival, histological structure of key organs, and tissue residue levels in *Archachatina marginata*. Therefore, sub-adult (under 30 g) *Archachatina marginata* snails were exposed for 10 weeks, once a week to field-relevant concentrations of glyphosate (0.5 g/L), metalaxyl (3.3 g/L), and cypermethrin (2 g/L), while the control group was exposed with tap water. The experiment was repeated four times. Survival and body weight were recorded weekly. Post exposure, tissue residues were analyzed by GC-MS, and histological examinations of the kidney and ovo-testis (snails' gonad) were performed. The result showed no significant differences in the survival or external morphology of exposed snails. However, deeper statistical analyses revealed that snails exposed to metalaxyl had significantly lower final weights compared to all other groups with a mean loss of −8.7 g (−26.6%). A histological examination revealed visible alteration in the kidney and ovo-testis tissues of treated snails, though these changes could not be confirmed statistically. Moreover, pesticide residues were detected in the tissues of treated animals, with trace amounts of glyphosate and cypermethrin also found in control snails, likely due to prior contamination or cross-cage drift. In conclusion, sub-chronic exposure to field-used pesticides did not induce mortality in *Archachatina marginata* but did affect growth and tissue integrity, especially under metalaxyl exposure. These findings raise concerns about sub-lethal toxicity and food safety risks and support the use of *Archachatina marginata* as a bioindicator in pesticide-exposed environments.

## 1. Introduction

Cameroon has four agroecological zones including rainforest monomodal area known to be predominantly agricultural area. Industrial plantations can be found: palm oil, rubber, coffee, cocoa, etc. Made up of agrarian population, varieties of crops are cultivated for both family nutrition and to supply urban centers. These crops range from annual to perennial and include leafy vegetables, root/tuber, plantain/banana, oil palm, etc. To face the increasing demand of the growing population in terms of food, farmers have turned to pesticide use in order to fight against pest that will besides contribute to improved crop production [1–3]. A survey carried out on pesticide application in Cameroon has pointed out those that are widespread [4]. Among those identified are glyphosate, an herbicide, cypermethrin, an insecticide, and metalaxyl, a fungicide [4]. Conversely, farmers usually have few information concerning the safe use of pesticides [4]. Indeed, farmers make a hazardous mix of the pesticides and the concentrations are usually higher than what is recommended. Many of them do not have adequate protective clothing (mask, impermeable boots, trousers and long sleeve shirts, etc.) while spraying pesticides [4]. Pesticides have not only certainly increased agricultural production but have also led to pollution of the environment and suspected causing harmful effect on the health of exposed population [5–14]. Besides, the rainforest area possesses factors that favor the growth of land snails [15, 16]. The widespread variety in the area is the *Achatinidae* family, also known as African giant snails [17]. Their consumption in the area is common [18], and according to sellers, *Archachatina marginata* meat is the most appreciated by consumers. These snails are found on the soil of farms since they consume fallen fruits and leaves [15, 16]. Studies showed that snail meat offers several nutritional benefits (rich in protein and low in fat and carbohydrate) [19–21].

The impact of environmental pollutants, including pesticides, has been widely studied in developed countries where regulations and monitoring systems are more established. Most of these research studies have focused on aquatic invertebrate, but over the years, terrestrial invertebrates particularly land snails have also gained attention as useful bioindicators of soil pollution. In Europe, the work of De Vaufleury and colleagues has shown how specie like *Helix aspersa* can accumulate toxicants and reflect the health of their environment [22–24]. Similarly, Al-Alam and Baroudi have examined pesticide residues in terrestrial snails and highlighted potential risks to human consumers [25].

In sub-Saharan Africa, ecotoxicological studies on pesticide impact on snail are still limited. Most research related to this topic tends to focus on socio-economic impacts, agricultural productivity, or human health perception [5–14]. There are only few studies that specifically address how these chemicals affect local edible land snails. Notably, research conducted in Nigeria, Benin, and Côte d'Ivoire has suggested that snails from the *Achatinidae* family can accumulate pesticide residues and heavy metals that may serve as early warning indicators of environmental contamination [26–28].

In Cameroon, the available data are even more limited. To our knowledge, only one published study has assessed that the wild-collected *Achatinidae* snail flesh might be contaminated with microorganism and fungi toxins and could be potential biohazards to human health [29]. Knowing the increasing consumption of snail meat in Cameroon [18], this is especially relevant as small-scale snail farming becomes more common and often takes place near potentially pesticide-polluted agricultural plots. Indeed, our previous work found detectable levels of glyphosate and metalaxyl in wild-collected *Archachatina marginata*, and we observed organ-specific histological changes in exposed animals [30]. Given this context, the present study was designed to investigate the sub-chronic effects of glyphosate, metalaxyl, and cypermethrin, three commonly used pesticides in Cameroon under conditions similar to those found in backyard or semi-intensive snail farming. The objective was to assess whether such exposure could lead to residue accumulation, affect growth and survival, and cause tissue damage in *Archachatina marginata* snails. This work aims to contribute to the limited but growing body of evidence on environmental risks in African agro-ecosystems and to support better-informed decisions regarding food safety and pesticide regulation.

## 2. Materials and Methods

### 2.1. Animals

**Figure 1:**
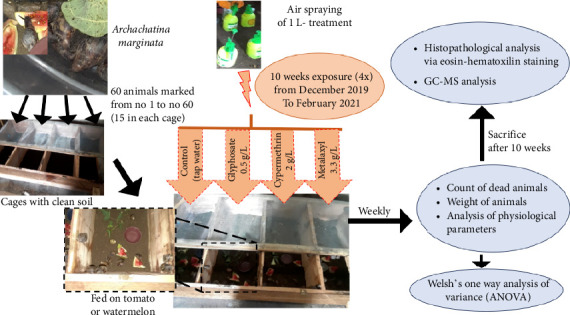
Schematic overview of the experimental setup. *Archachatina marginata* snails were divided into four treatments groups and exposed weekly for 10 weeks to three commonly used Cameroonian pesticides (glyphosate, metalaxyl, and cypermethrin) or tap water (control). The experiment was repeated four times with 15 snails per group in replicates 1–3 and 10 in replicate four due to supply constraints. Monitored parameters were general appearance, weight, and survival. Post exposure, snails were processed for histological and residue analyses.

*Archachatina marginata* snails were obtained from a local farm in Kribi (South, Cameroon) and divided into four groups housed in separated 32 m^2^ wooden cages with clean soil and a plastic mesh cover (Figure 1). Each group included 15 snails in the first three experiments and 10 in the fourth due to supply limitations. *According to Cobbinah and colleagues, Archachatina marginata* snails can grow up to 20 cm in length and weigh as much as 500 g [31]. However, they typically reach sexual maturity at around 1 year of age, when they weigh approximately 100–125 g [31]. The individuals used in this study, with an average weight of about 30 g, were therefore considered sub-adults or juveniles, likely between 4 and 6 months old. Animals were fed daily with four medium tomatoes or two slices of watermelon, while tap water was provided through a plastic bowl. The remaining food was removed daily, and the soil at the bottom of the cage was cleaned weekly to prevent any disease contamination.

### 2.2. Pesticide Exposure

Ten/fifteen *Archachatina marginata* snails per group were exposed primarily through cutaneous contact using a 1 L air sprayer. However, digestive exposure could not be excluded, as both the snails and their food remained inside the enclosures during the spraying process. Exposure was performed with three commonly used pesticides: glyphosate 0.5 g/L (Arysta Life Sciences, Noguères, France), metalaxyl 3.3 g/L (Arysta Lifesciences, Noguères, France), and cypermethrin 2 g/L (AFCOTT Cam sarl, Garoua, Cameroon). The applied concentrations reflected typical field application rates used by farmers. The control group was exposed to tap water under the same conditions. Pesticide application was performed over and within the designated compartment of each animal's cage. Exposure was done once a week for 10 consecutive weeks (2.5 months), and the full experimental cycle was repeated four times between December 2019 and February 2021. The frequency of exposure once per week during the cultivation season reflects common pesticide application practices in the region, although the overall duration may vary depending on the specific crop cultivated. Nevertheless, the concentrations used correspond to actual doses commonly applied by local farmers. This design allowed for the assessment of cumulative impacts under realistic yet intensified exposure conditions.

### 2.3. Evaluation of Animal External Physical Aspect and Survival

External physical aspect (shell condition and the soft part) and survival through the number of dead animals were monitored once a week before exposure to various treatments. Snails' weights were measured weekly using a precision scale to track weight changes over time in each treatment group.

### 2.4. Histology

Three snails from each exposed groups were randomly chosen for histological studies. Snails were starved 48 h to ensure the digestive tracks were void of ingested food. Animals were sacrificed into boiled–recooled water and directly put in the fixative solution formaldehyde 4%. Dissection was done according to Low et al.'s protocol [32]. Snail soft part was separated from the shell; then, ovo-testis (hermaphrodite gonad) and kidney were excised out. Furthermore, the excised organs were immersed in subsequent steps from 70% to 100% ethynyl alcohol for dehydration, followed by xylene incubation used as a clearing agent. The organs were embedded in paraffin; 2–5 μm thickness sagittal sections were done using a rotary microtome and stained with hematoxylin and eosin. Microphotographs of the sections were taken using an OMAX 40X-2500X LED digital trinocular microscope with a USB digital camera (Irvine, California, USA) and analyzed with ImageJ 1.53a software.

### 2.5. Extraction and Clean-Up for Gas Chromatography–Mass Spectrometry (GC-MS)

All the experiments of GC-MS were carried out at the Faculty of Agriculture, Damanhour University, Egypt. Soft tissues from 7 to 10 remaining snails per treatment group across four experiments were freshly dissected, gently blotted to remove excess moisture, and weighed. To minimize mucus interference during homogenization, samples were rinsed gently. The mix of 10 g of four snails' soft part were put together and then crushed using a chilled mortar and pestle prior to extraction for each group. Then, 10 mL of acetonitrile, 2 g of MgSO_4_, and 0.5 g of NaCl were then added to the tissue. The mixture was vortexed for 1 minute and shaken for 15 min. Then, the samples were centrifuged at 3000 rpm for 5 min. One milliliter of the supernatant was mixed with 100 mg of primary secondary amine (PSA) and 400 mg of MgSO4, vortexed for 2 min, and centrifuged as described previously. The solvent was filtered through a 0.25 μm thickness filter disk and used for GC-MS determination [33].

### 2.6. GC-MS Determination

For GC-MS determination, Agilent Technologies 7890 GC system-coupled with MS-5977A MSD, Japan ,was used. The GC-MS instrument with electron impact (EI) ionization, autosampler (AS), and computerized instrument control/data collection was used. Two microliters were used for injection volume as a spitless mode at 250°C. The analytical column (30 m, 0.25 mm id, and 0.25 μm thickness of 5% phenyl methyl polysiloxane) was used, and helium was used as a carrier gas at a rate of 1 mL/min. The temperature program was started at 100°C and ramped to 280°C at a rate of 10°C/min. The software program was used to estimate the output data.

### 2.7. Analytical Method Validation

The laboratory glassware was soaked for 12 h in acid solution, then washed carefully and purged with distilled water and acetone, and dried before use. All chemicals used were of analytical grade. The limits of detection (LODs) ranged from 0.005 to 1.0 ng/mL for the examined pesticides. Recovery experiments were performed to evaluate the efficiency of the extraction and cleanup procedures. Blank snail tissue samples were spiked with known concentrations of glyphosate, metalaxyl, and cypermethrin and then subjected to the same analytical procedure as the test samples. The recovery rates ranged from 94.0% to 95.5%, indicating a reliable extraction efficiency. Calibration curves were prepared with regression coefficients (*R*^2^) ≥ 0.995. All analyses were performed twice (in duplicate) to ensure reproducibility.

### 2.8. Statistics

Means of weights and animals' survival as well as an estimated vacuole quantity within the eosin–hematoxylin-stained kidney were compared. The comparison was performed using Welsh's one-way analysis of variance (ANOVA) with GraphPad Prism 9 software. A *p* value *p* ≤ 0.05 was considered significant. Moreover, we performed a Tukey HSD post hoc test to better understand whether differences between treatment groups were statistically significant. The mean of pesticide concentrations was determined by quantifying peak areas generated during GC-MS analysis. These areas were automatically integrated and quantified by the instrument's software using calibration curves established from certified reference standards. The means were compared using the *t* Student test and considered significant with the *p* value *p* ≤ 0.05.

### 2.9. Ethics Statement

All experiments have been conducted in accordance with the current scientific bioethics law of Cameroon. All organisms unexposed to toxicants (i.e., unused and untested) were given as food.

## 3. Results

### 3.1. Evaluation of Animal External Physical Aspect and Survival

Snails treated with metalaxyl showed signs of fatigue about 2 days after exposure, such as reduced movement and leaving food uneaten. However, no visible differences were observed in the shell or soft body appearance between treated and control groups (tap water).

**Figure 2:**
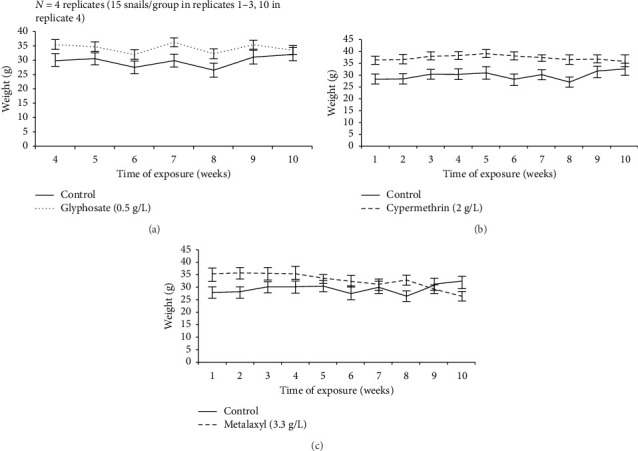
Effect of pesticide versus tap water exposure on the weekly mean body weight of *Archachatina marginata* over a 10-week period. Data represent mean ± standard error of the mean (SEM) from four independent experiments. Final weights of metalaxyl-exposed snails were significantly lower than those of all other groups (Tukey HSD, *p* < 0.001), although no overall group differences were detected by Welch's one-way ANOVA.

**Figure 3:**
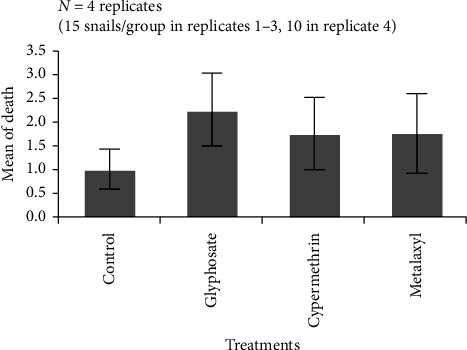
Effect of pesticide exposure on *Archachatina marginata* survival over a 10-week period. The mean number of deceased snails per group was recorded across four independent replicates (*N* = 4) ± SEM. Each group included 15 snails per replicate, except for the fourth, which included 10 snails due to availability constraints. No statistically significant differences in mortality were observed between pesticide-treated and control groups based on Welch's one-way ANOVA (*p* > 0.05).

ANOVA analysis showed no significant differences in the overall weight between groups during the 10-week period (Figure 2). Still, weight trends varied: the control, glyphosate, and cypermethrin groups gained +4.2 g (+14.3%), +2.0 g (+5.9%), and +3.9 g (+11.1%), respectively (Table 1). In contrast, snails exposed to metalaxyl lost −8.7 g (−26.6%) (Table 1). Over time, metalaxyl exposure led to a steady weight loss, while the other groups showed consistent weight gain. Post hoc analysis (Tukey HSD) confirmed that metalaxyl-treated snails had significantly lower final weights compared to all other groups (*p* < 0.001). Survival rates did not differ significantly between treatments (ANOVA, *p* ≤ 0.05) (Figure 3).

### 3.2. Effect of Pesticide Exposure on *Archachatina marginata* Ovo-Testis Structure

**Figure 4:**
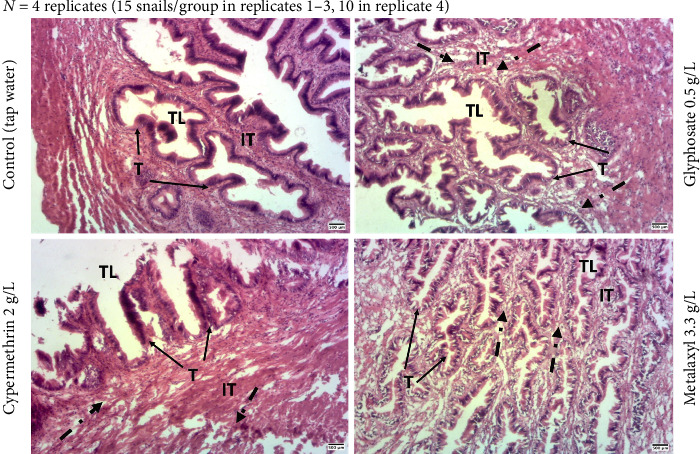
Histological sections of the ovo-testis in *Archachatina marginata* following subchronic pesticide exposure. Representative eosin–hematoxylin–stained ovo-testis sections from three randomly selected snails per group, drawn from four independent experimental replicates (*N* = 4). Each group included 15 snails in the first three replicates and 10 in the fourth due to availability constraints. Snails were exposed to glyphosate, metalaxyl, or cypermethrin and compared to a control group (tap water). While no statistically significant differences were observed, morphological alterations in the interstitial tissue were apparent in pesticide-exposed groups. Dashed arrows indicate regions of altered interstitial tissue. Abbreviations: IT = interstitial tissue; T = tubule; TL = tubule lumen. Scale bar = 500 μm (40x magnification).

Histological analysis of the *Archachatina marginata* hermaphroditic gonad (ovo-testis) was conducted to assess whether whole-body pesticide exposure might affect its structural integrity. Although no statistically significant differences were confirmed, ovo-testis sections from exposed snails occasionally exhibited the interstitial tissue with an altered appearance compared to controls (Figure 4).

### 3.3. Effect of Pesticide Exposure on *Archachatina marginata* Kidney Structure

**Figure 5:**
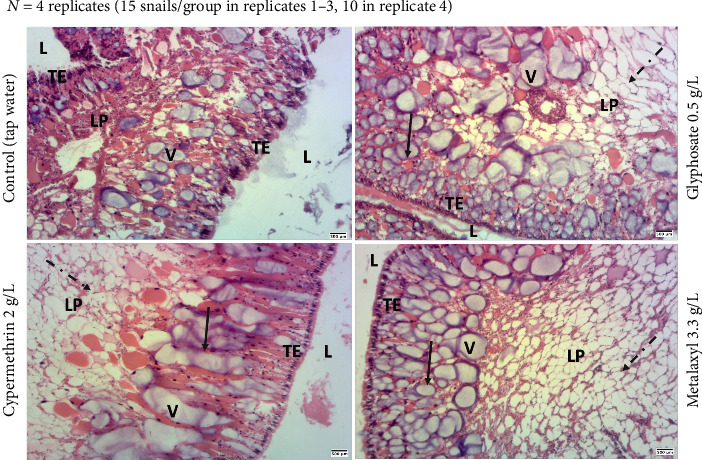
Histological sections of the kidney in *Archachatina marginata* after subchronic pesticide exposure. Representative eosin–hematoxylin–stained sections from three randomly selected snails per group, drawn from four independent experimental replicates (*N* = 4). Each group contained 15 snails in the first three replicates and 10 snails in the fourth due to availability constraints. Snails were exposed to glyphosate, metalaxyl, or cypermethrin and compared to a control group (tap water). Observed alterations include apparent disorganization of the lamina propria and enlarged vacuoles in pesticide-treated snails compared to controls. Solid arrows indicate enlarged vacuoles; dashed arrows indicate disrupted lamina propria. Abbreviations: V = vacuoles; LP = lamina propria; TE = transitional epithelium. Scale bar = 500 μm (40x magnification).

Histology of the kidney of *Archachatina marginata* snail was performed to determine whether direct pesticide exposure on the whole snail could disrupt this structure. Although vacuole counts did not differ significantly between groups (Table 2), some exposed individuals showed morphological alterations such as enlarged vacuoles and mild disorganization of the lamina propria compared to controls (Figure 5).

### 3.4. Pesticide Measurements on Snail Soft Part

**Figure 6:**
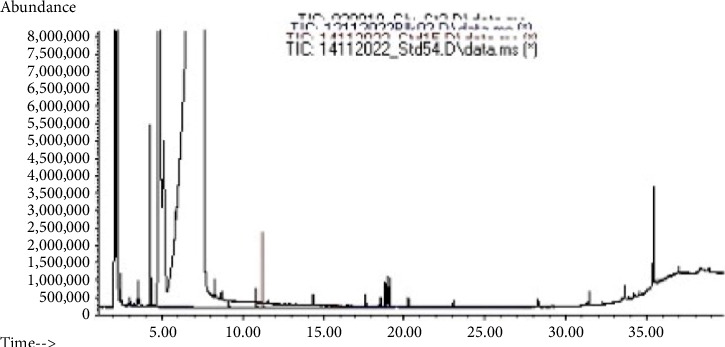
Representative GC-MS chromatogram showing pesticide residues detected in homogenized soft tissues of *Archachatina marginata* following subchronic exposure. Each chromatographic peak corresponds to the mass spectra of individual pesticide and/or their degradation products or isomers, as identified by the instrument software based on retention times. Tissue samples were pooled from four independent replicates (*N* = 4) for analysis.

As shown in Table 3 and Figure 6, pesticides were found in some treated snails' flesh when compared to the controls. However, cypermethrin and glyphosate were also found in the control contrary to metalaxyl that was undetectable (Table 3). However, the pesticide values measured were within the range of lethal dose for 50% of animals tested (LD_50_) for contextual reference and maximum residue levels (MRLs) inside food recommended by Food and Agriculture Organization (FAO) and World Health Organization (WHO) (Table 3).

## 4. Discussion

This study evaluated whether sub-chronic exposure to commonly used Cameroonian pesticides under semi-farming conditions affects growth, survival, residue levels, and tissue integrity in *Archachatina marginata*. Building on our previous finding, one of our hypotheses was that exposure to these pesticides could negatively affect *Archachatina marginata* snail health [30]. As a result, no significant difference was observed in the animal shell condition, external soft body appearance, or survival rate between treatment groups. Similarly, ANOVA revealed no significant differences in the mean weight among groups. However, post hoc analysis using Tukey HSD showed that snails exposed to metalaxyl had a significantly lower final weight, with a mean loss of −8.7 g (−26.6%) compared to all other groups. This weight loss was consistent over time, suggesting a cumulative effect that may possibly be linked to reduced metabolism or feeding activity. This interpretation is supported by behavioral observations: snails exposed to metalaxyl showed signs of fatigue approximately 2 days after metalaxyl exposure, including reduced mobility and incomplete food consumption, compared to those in the other treatment groups. In contrary, snails exposed to cypermethrin and glyphosate both gained weight. The cypermethrin group showed greater variability, despite starting with lower weights gradually gained over time, resulting in a mean weight increase of +11.1%. The glyphosate group showed more modest effect by steady growth (+5.9%). Control animals showed the highest weight increase (+14.3%), confirming stable and good health under the study conditions. Despite the absence of mortality differences, the observed weight reduction in the metalaxyl group and the statistically confirmed differences in the final weight highlight the importance of monitoring growth as a sensitive biomarker of pesticide stress. These findings align with other studies that also reported snails' weight impacted by pesticides. For example, Ogeleka et al. [38] showed in contrary weight reduction of *Archachatina marginata* after exposure to Grassate, a nonselective-glyphosate-based herbicide. This difference may be due to the higher concentrations of pesticides used in that study compared to the more field-representative doses applied here. Similarly, N'guessan et al. [28] showed that the weight and growth of *Achatina achatina* snails (another snail specie within *Achatinidae* snails' family) were inhibited in a dose-dependent manner following glyphosate exposure.

We also tested whether exposure to these pesticides has a deleterious effect on the structure of certain organs, namely, the ovo-testis and the kidney. Gonad and kidney are organs known to be sensible and be disrupted by various endocrine disruptors including pesticides. Indeed, studies have shown their disruption after exposure to pesticides in laboratory animals [35, 36, 39–41]. After histopathological analysis of the ovo-testis sections, there is absence of observable gametes in ovo-testis sections that may be attributed to the immature status of the snails used (estimated age around 4–6 months). Moreover, we found that animals exposed to pesticides showed a visible alteration of the hermaphroditic organ interstitial tissue. This result is in agreement with our previous study [42]. Moreover, we also found out that visible disorganization of the lamina propria, as well as vacuoles of the pesticide exposed-kidney sections, tends to be enlarged. These structural changes, while not statistically confirmed, may suggest early signs of tissue stress following exposure. As a future perspective, conducting reactive oxygen species (ROS) analysis would be valuable to better assess oxidative stress and further clarify the sublethal effects of pesticide exposure in snails. Moreover, it will be essential to determine whether the pesticide-induced structural disturbances of these organs could affect their functionality. Therefore, perspective for this part will be to measure reproductive hormones and kidney markers.

Chromatographic analysis was performed to detect pesticide residues in snail tissues, which is particularly relevant given that *Archachatina marginata* snails are widely consumed in the area. The row chromatograms showing distinct peaks for each compound were not archived and not made available by the external laboratory that conducted the GC-MS analysis. Consequently, detailed visualization of the individual mass spectra and retention times could not be included. Although the absence of row chromatograms limits the ability to present peak-level evidence, the analysis followed widely accepted analytical quality assurance protocols. As a result, the measured pesticide values can be considered reliable. Based on this analysis, glyphosate, cypermethrin, and metalaxyl were confirmed to be present in the flesh of the exposed snails. This indicates that these pesticides can be transferred to the snail tissues following subchronic exposure. These results are consistent with our previous study [30] and are further supported by other studies that have reported the presence of pesticides within the flesh of exposed snails [27, 42–45]. Unexpectedly, traces of glyphosate and cypermethrin but not metalaxyl were also detected in snails from the control group. This may be due to the absence of a baseline residue screening in both snails and tap water prior to the start of the experiment, representing a limitation in the study design. Moreover, the snails used were purchased from local snail farmers. However, it is known that due to limited supply, many small-scale farmers in Cameroon often collect snails from the wild to meet market demand. It is therefore plausible that some of the animals used in this study were already contaminated prior to the experiment. Additionally, the proximity of cages during pesticide application may have contributed to unintentional cross contamination via airborne drift. Interestingly, metalaxyl was not detected in control animals, which could reflect its lower volatility compared to glyphosate and cypermethrin. This interpretation is supported by findings from our earlier study where metalaxyl was not detected in snails collected from areas with low agricultural activity [30]. The concentrations of pesticide residues measured in exposed snails in this study were within the MRLs established by the WHO and FAO. For context, LD_50_ values in rodents were included to illustrate the relative toxicity of the tested compounds in animals, as specific LD_50_ data for *Archachatina marginata* or other terrestrial snails are scarce in the literature. However, studies in other snail species and invertebrates have reported LC_50_ values that offer relevant insights. Despite the relatively high concentrations of pesticides used in this study, glyphosate (0.5 g/L), cypermethrin (2 g/L), and metalaxyl (3.3 g/L), which exceed published LC_50_ values for aquatic invertebrates and freshwater snails, no statistically significant differences in survival were observed between the treated and control groups as mentioned earlier. These exposure concentrations reflect actual doses commonly used by farmers in Cameroon, making them representative of real-world agricultural practices. For instance, cypermethrin LC_50_ values are reported to vary seasonally, ranging from 10.39 mg/L in May to 65.84 mg/L in January in *Lymnaea acuminata* [46] and 44.59 mg/L in *Chilina parchappii* [47], while the exposure dose used here (2000 mg/L) is far higher. Similarly, the exposure concentration of metalaxyl (3300 mg/L) greatly exceeds 48 h LC_50_ values reported for *Daphnia magna, an aquatic invertebrate* (racemic metalaxyl, 41.9 mg/L and R-metalaxyl, 51.5 mg/L) [48], and glyphosate (500 mg/L) was applied at levels surpassing 96-h LC_50_ in *Pomacea canaliculata* (175 mg/L) [49]. Although the pesticide concentrations used reflected actual field practices and exceeded known toxicity thresholds for some aquatic species, they did not cause acute effects on survival in *Archachatina marginata*. This may reflect lower absorption or higher tolerance under terrestrial conditions or due to species physiological differences. However, the presence of pesticide residues in snail tissues and observed histological changes suggests potential sublethal effects. Importantly, regulatory residue limits do not ensure safety, especially considering possible bioaccumulation and frequent consumption. Thus, even low-level contamination could pose long-term health risks.

## 5. Conclusion

Due to international regulations on pesticide residues in export crops such as coffee and cocoa, Cameroonian government has been promoting reduced pesticide use in agriculture. It is within this context that the present study was conducted, aiming to evaluate under controlled conditions the impact of three widely used pesticides. The present study enlightens that subchronic exposure to field-used pesticides did not induce mortality in *A. marginata* but did affect growth, especially under metalaxyl exposure. As well as histological alterations in the kidney and gonad further confirm potential sublethal toxicity. Moreover, the detection of pesticide residues in edible tissues, even within regulatory limits, points to a potential health risk due to bioaccumulation and repeated dietary exposure. These results support the use of *Archachatina marginata* as a sentinel species for monitoring terrestrial pesticide contamination and highlight the need for further research on chronic and cumulative effects of pesticide exposure in food-chain organisms. As a precautionary measure, we recommend that stakeholders in the snail sector prioritize farmed snails over those collected in pesticide-treated fields. Looking ahead, future research will expand this work by assessing the impacts of other emerging environmental contaminants, particularly plastic-derived pollutants, which are also increasingly common in the region.

## Figures and Tables

**Table 1:** Summary of weight variation in *Archachatina marginata* following 10-week pesticide exposure.

Treatment	Weight mean in week 1 (g)	Weight mean in week 10 (g)	Weight means from week (1–10) (g)	Weight change	Percentage of change
Control (tap water)	28	32.2	29.5 ± 0.5	4.2	14.3
Glyphosate	31.7	33.8	34.1 ± 0.5	2	5.9
Cypermethrin	31.7	35.6	34.8 ± 0.5	3.9	11.1
Metalaxyl	35.3	26.6	32.8 ± 0.9	−8.7	−26.6

*Note:* Values are the mean of *Archachatina marginata* snails' weights measured weekly from four independent experiments following exposure to tap water (control), glyphosate (0.5 g/L), metalaxyl (3.3 g/L), or cypermethrin (2 g/L) over a 10-weeks period. Weight changes are expressed in grams and as percentage variation from week 1 to week 10. Statistical comparison using the Tukey HSD post hoc test revealed that metalaxyl-exposed snails had significantly lower final weights compared to all other groups (*p* < 0.001).

**Table 2:** Quantification of vacuoles in kidney histological sections of *Archachatina marginata* after subchronic pesticide exposure.

Treatments	Vacuole-estimated values
Control	105.9 ± 19.6
Glyphosate	137.9 ± 33.4
Cypermethrin	101.4 ± 11.6
Metalaxyl	133.4 ± 22

*Note:* Data represent the mean ± SEM for vacuole counts in eosin–hematoxylin–stained kidney sections. Values were compared across treatment groups using Welch's one-way ANOVA. Differences were considered statistically significant at *p* ≤ 0.05.

**Table 3:** Detected pesticide levels in the snail tissue compared with regulatory MRLs and rodent LD_50_ values (for contextual reference): pesticide residue level (μg/g tissue) in whole-body homogenate of exposed *Archachatina marginata* snails.

**Treatment**	**Mean of pesticide measured values**	**LD_50_ (measured in rodents)**	**MRL [34]**	

Control (tap water)	0.343 ± 0.26			*p*=0.222
Glyphosate	2.134 ± 1.5	1.6 ∗ 10^3^ [35]	0.55–500	

Control (tap water)	5.2265 ± 3.3			*p*=0.12
Cypermethrin	12.2715 ± 1.5	0.2–4 ∗ 10^3^ [36]	0.01–30	

Control (tap water)	ND			
Metalaxyl	7.8055 ± 0.9	0.669 ∗ 10^3^ [37]	0.1–10	

*Note:* Pesticide residues detected in *Archachatina marginata* tissues compared to regulatory MRLs and rodent LD_50_ values (for contextual reference). Mean pesticide concentrations (μg/g tissue) were measured using GC-MS analysis in pooled whole-body homogenates from exposed snails of four independent replicates (*N* = 4). Values represent mean ± SEM and were compared using Student's *t*-test (*p* ≤ 0.05). For comparison, regulatory MRLs (FAO/WHO) and LD_50_ values in rodents are shown; both were converted to μg/g for consistency. ND = not detected (below detection threshold), LD_50_ = estimated dose causing 50% mortality in test animals, and MRL = maximum residue limit set for food safety.

## Data Availability

All data generated or analyzed during this study are included in this article as supporting data.

## Supporting Information

Supporting Information 1 S1: Row data of morphology analysis. Weights of *Archachatina marginata* snail were measured weekly for 10 weeks, which were repeated 4 times. Then, they were analyzed statistically using Welsh's one-way analysis of variance with the *p*≤0.05 (ANOVA).

Supporting Information 2 S2: Row data of mass spectrometry analysis. The values showed in the table are the mean of measured values coupled with their standard error of the mean (SEM) obtained after the analysis of pesticide peaks coming from the homogenate of exposed snails' soft part by the software of the GC-MS instrument.

Supporting Information 3 S3: Row data of histological analysis. Microphotographs of the ovo-testis and kidney that were excised out from the collected *Archachatina marginata* snails, sectioned and stained with eosin–hematoxylin.

Supporting Information 4 S4: Row data of vacuole analysis. Estimation of vacuole quantity was performed within the eosin–hematoxylin–stained kidney of exposed animals compared together using Welsh's one-way analysis of variance (ANOVA). *p* ≤ 0.05 was considered significant.
