# Supplementary material for: Sublethal Effects of Cameroon Field-Used Pesticides on Growth and Organ Health in Archachatina marginata
Source: J Toxicol. 2025 Sep 12;2025:6365547. doi: 10.1155/jt/6365547 (PMC12449114; doi:10.1155/jt/6365547)
Supplement: Supporting Information 3 — S3: Row data of histological analysis. Microphotographs of the ovo-testis and kidney that were excised out from the collected Archachatina marginata snails, sectioned and stained with eosin–hematoxylin. [file 6365547.f3.pdf]

Ovo-testis Control

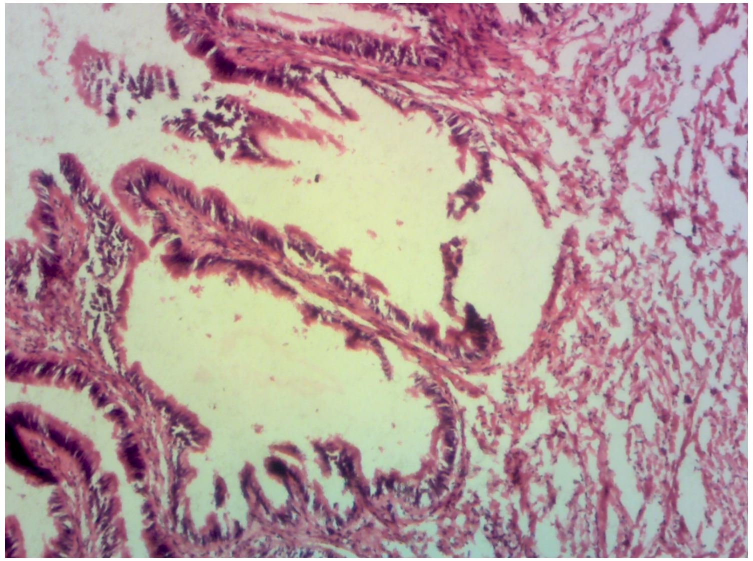

Section 1

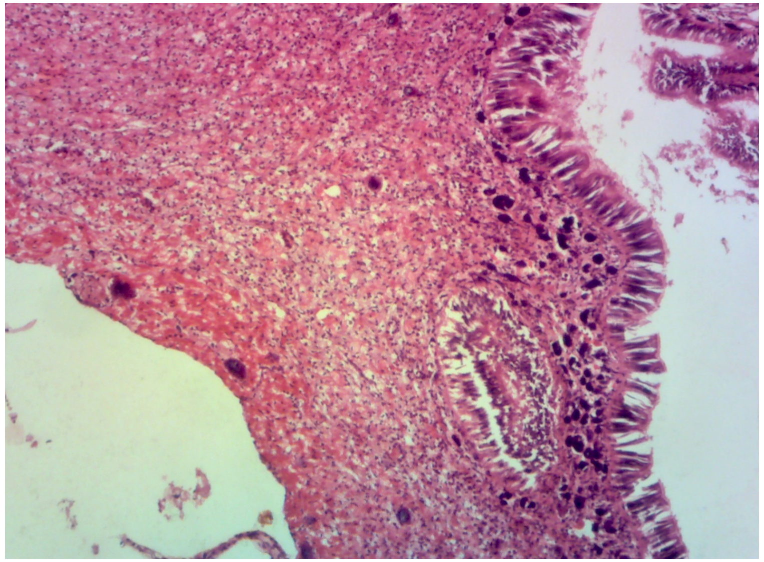

Section 2

Snail 1

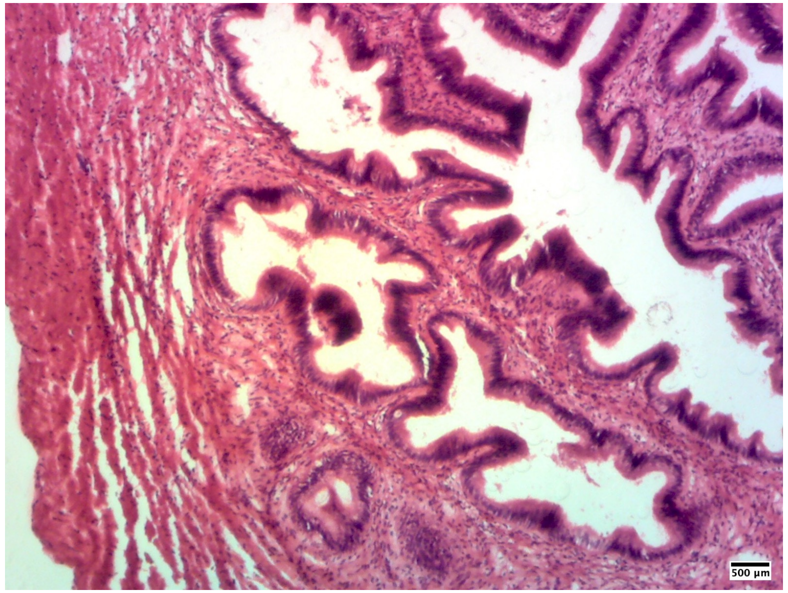

Section 1

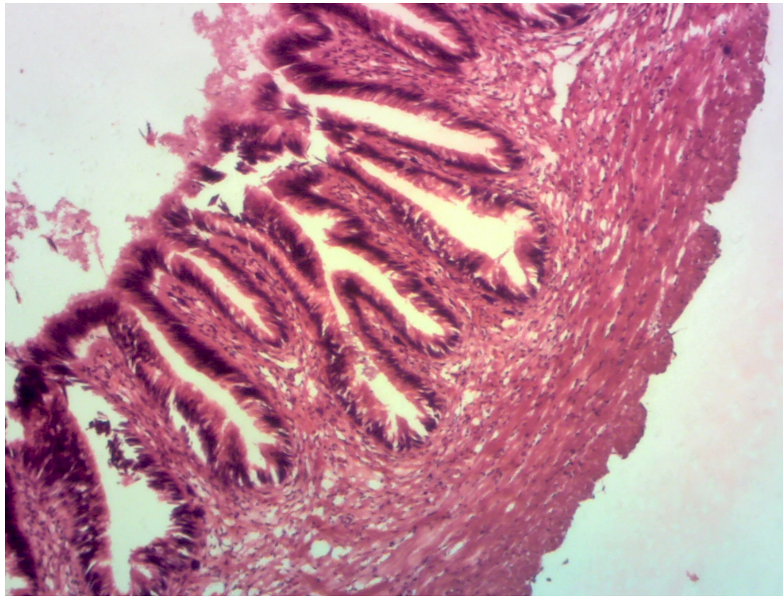

Section 2

Snail 2

Kidney Control

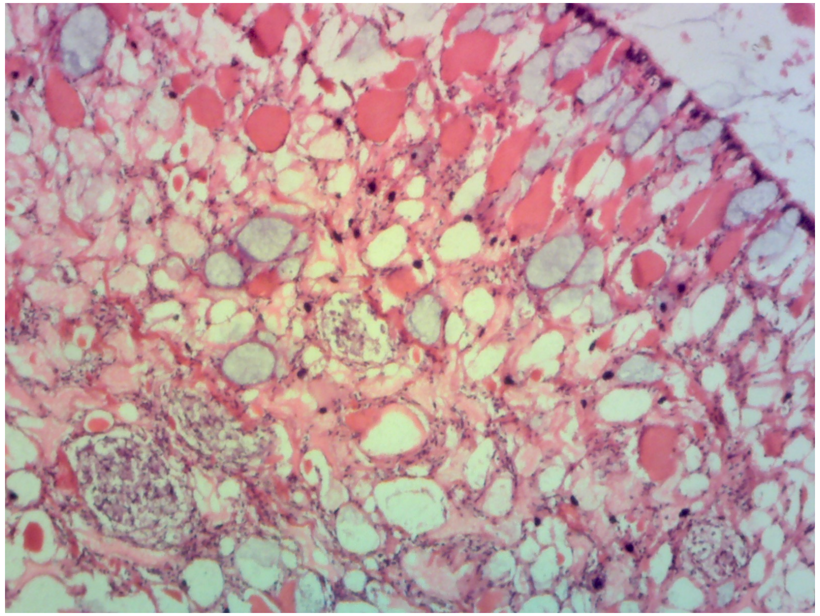

Section 1

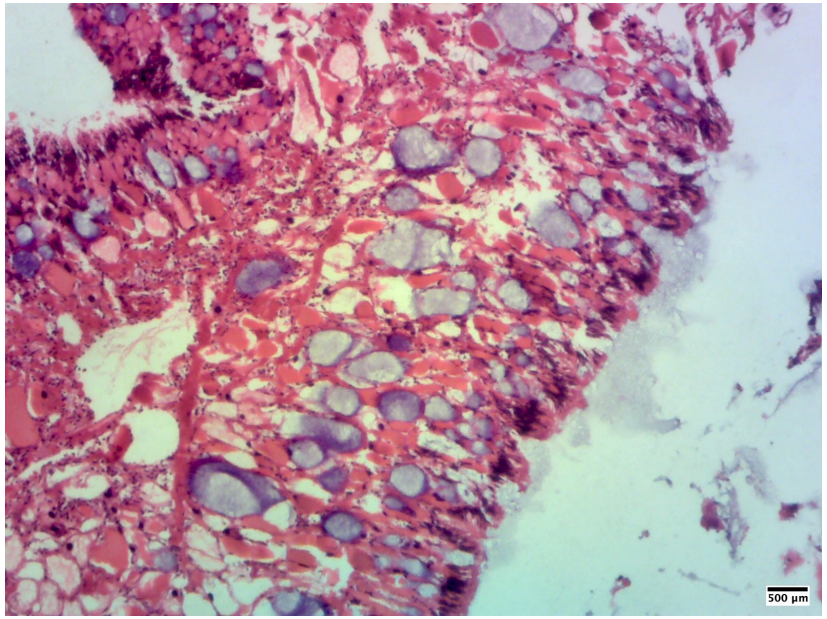

Section 2

Snail 1

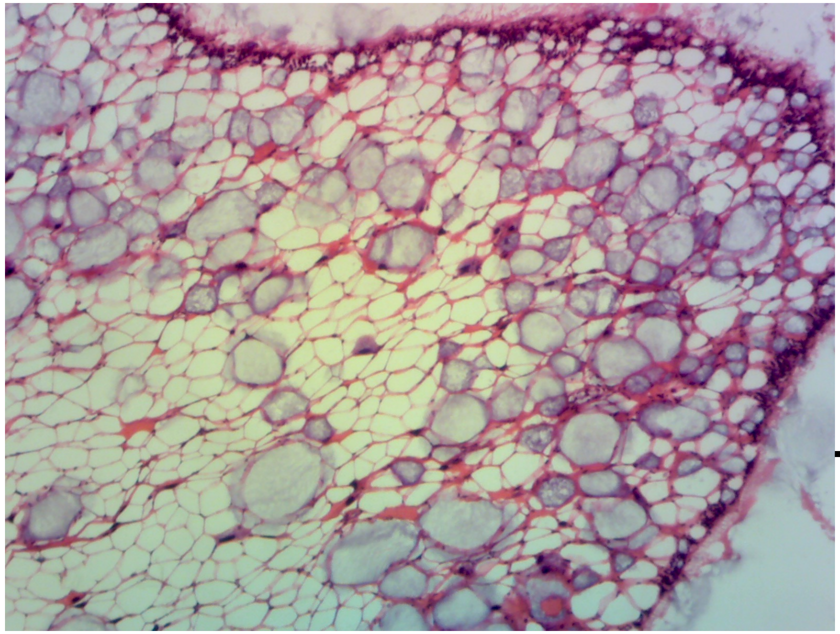

Section 1

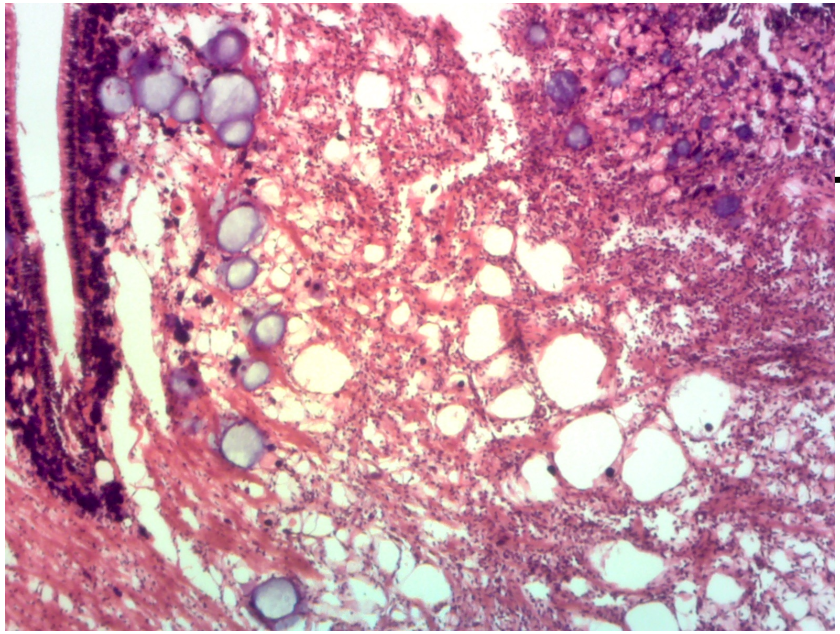

Section 2

Snail 2

Ovo-testis
Glyphosate

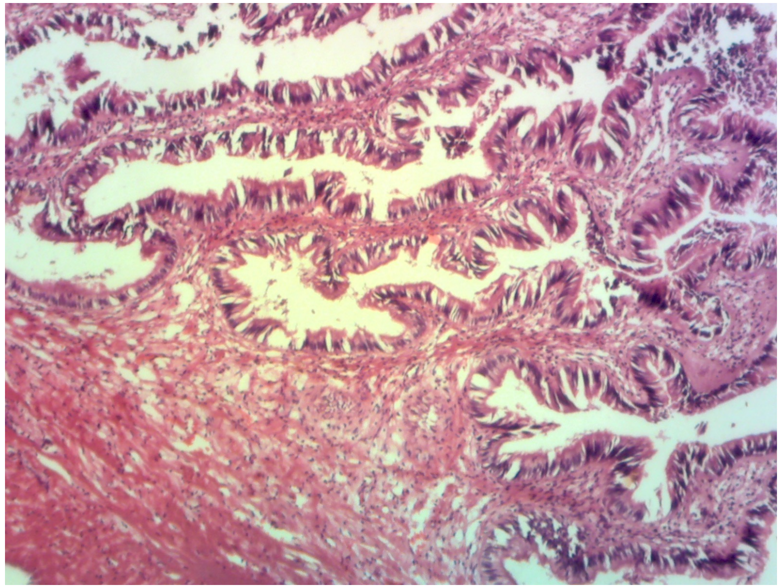

Section 1

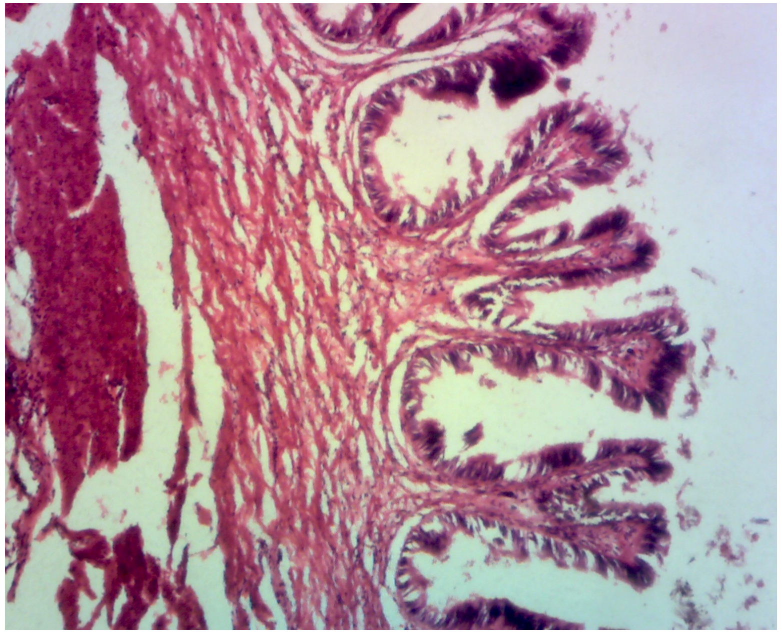

Section 2

Snail 1

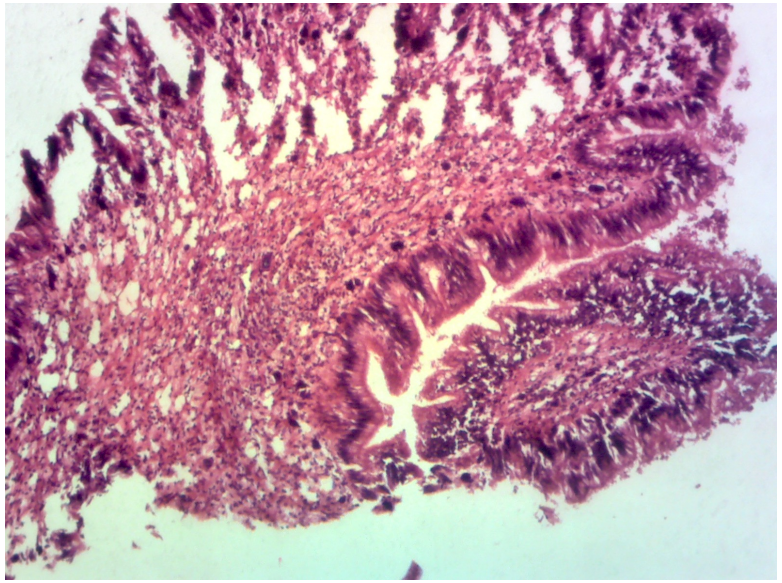

Section 1

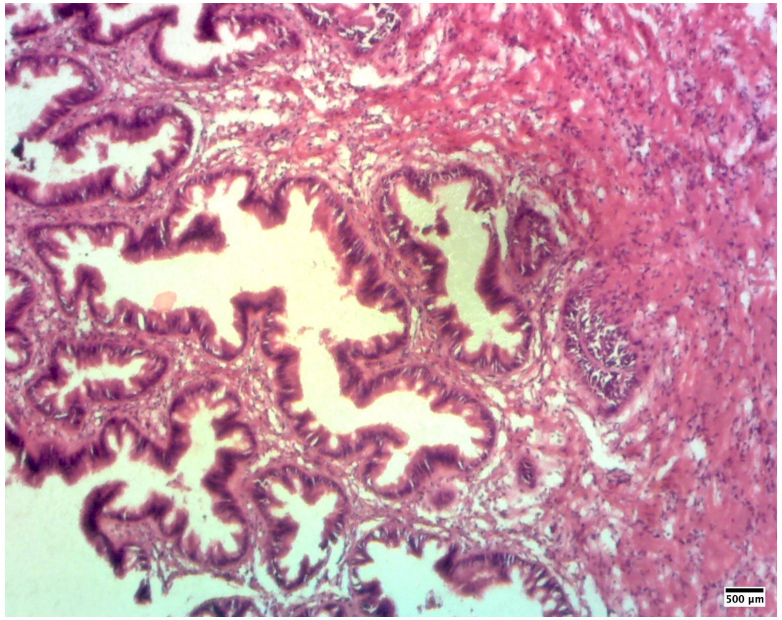

Section 2

Snail 2

Kidney Glyphosate

Snail 1

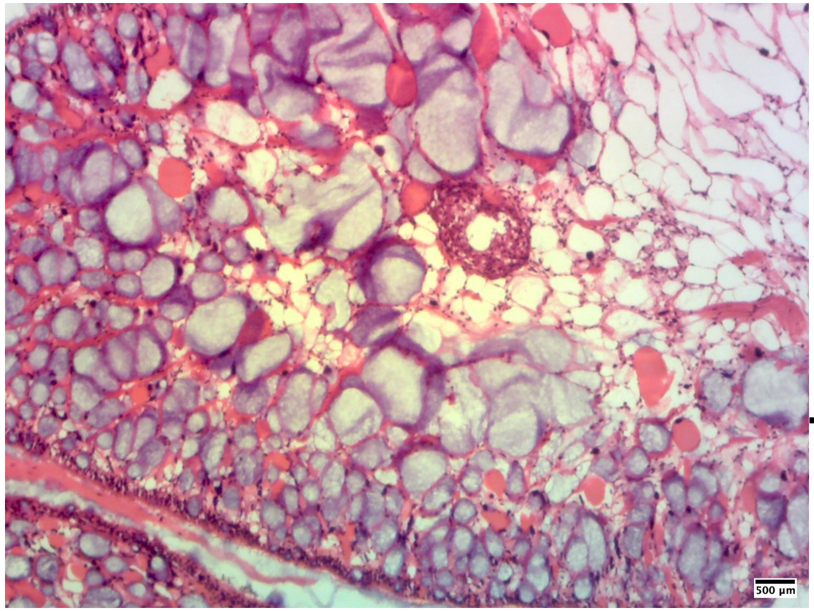

Section 1

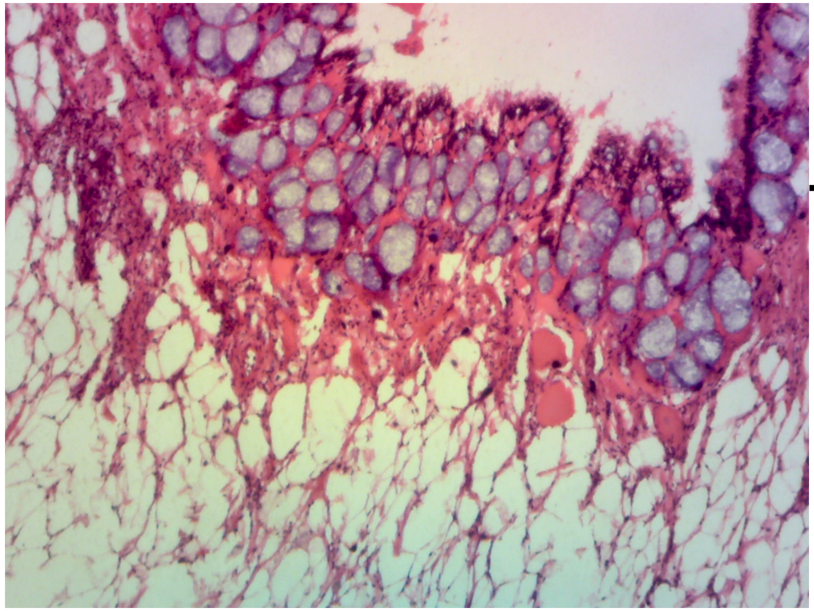

Section 2

Snail 2

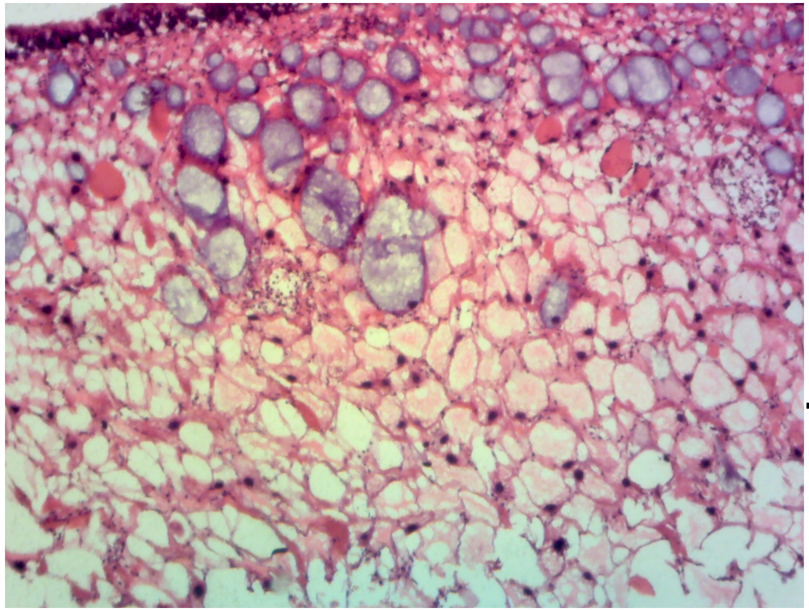

Section 1

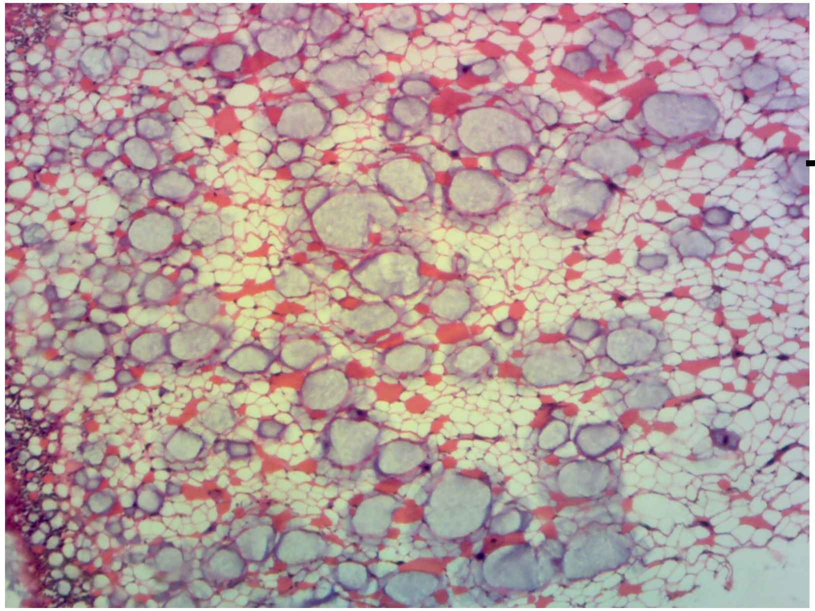

Section 2

Ovo-testis
Cypermethrin

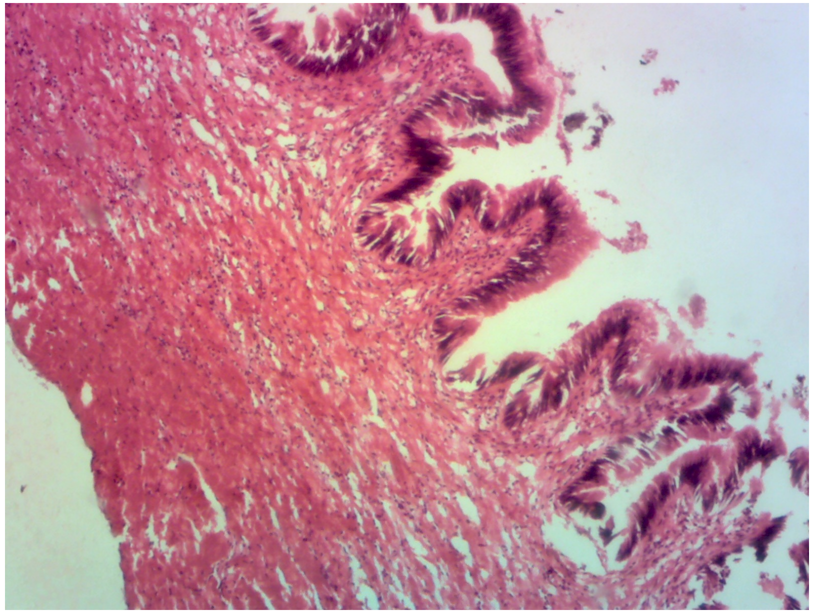

Section 1

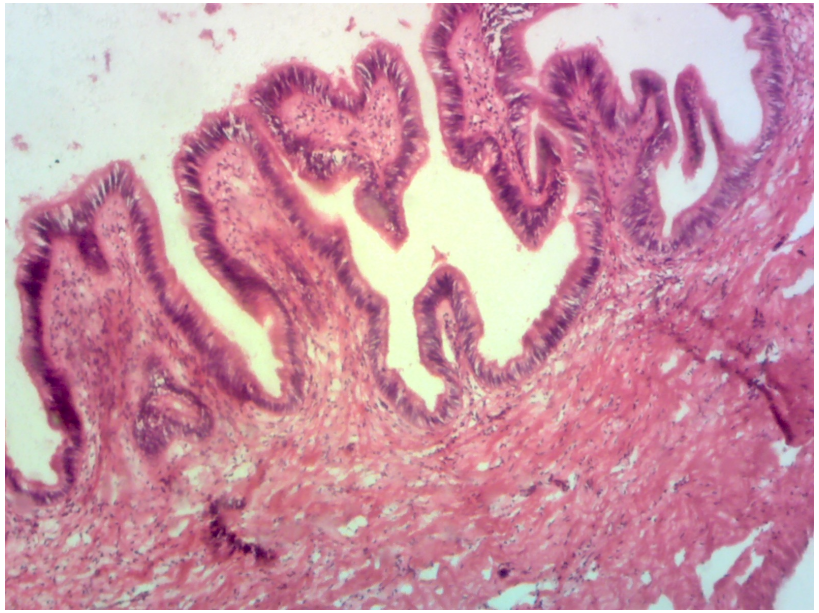

Section 2

Snail 1

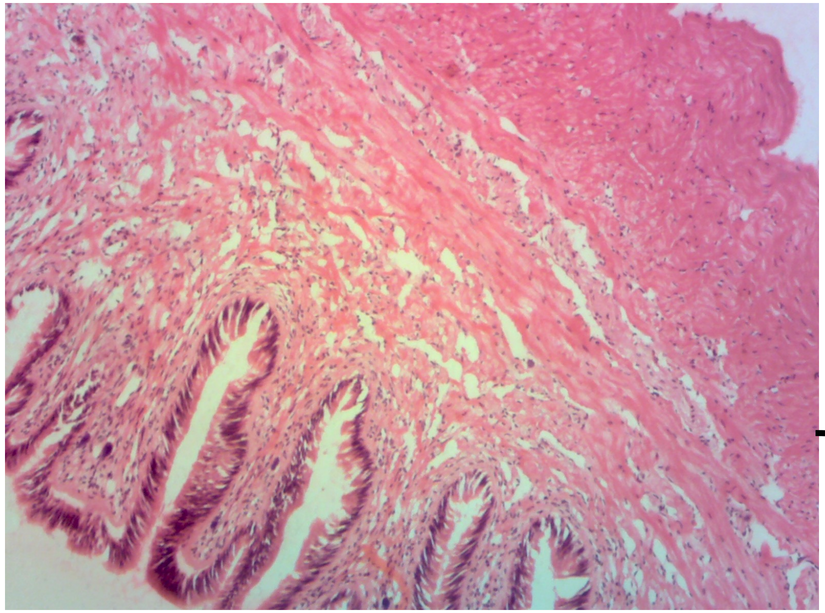

Section 1

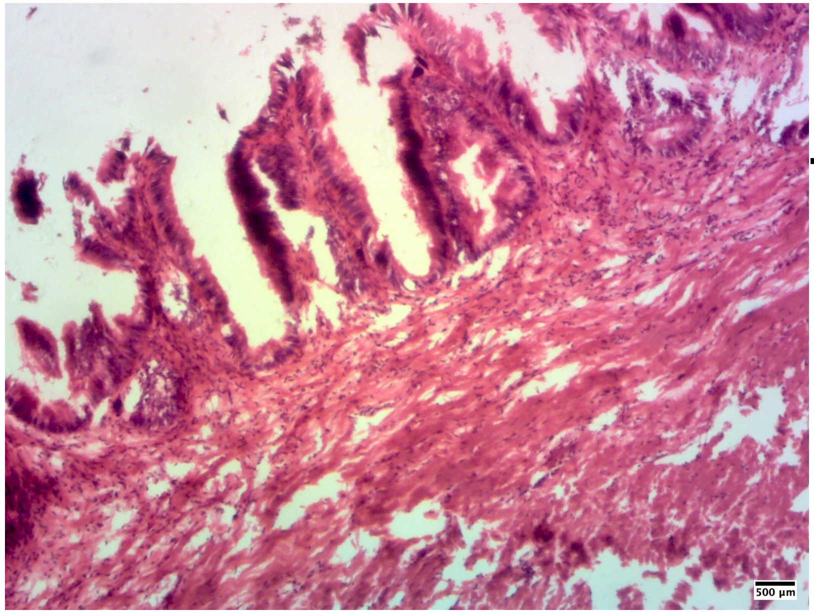

Section 2

Snail 2

Kidney Cypermethrin

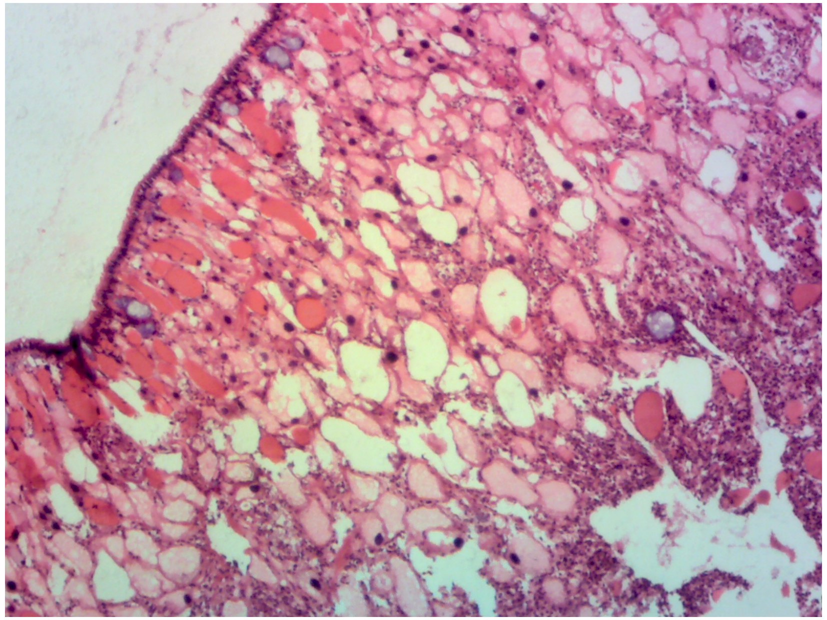

Section 1

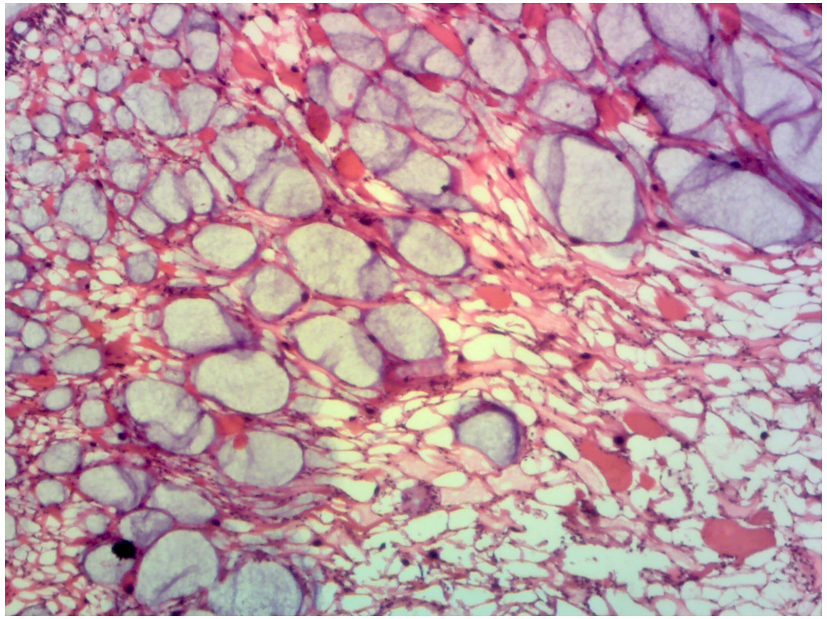

Section 2

Snail 1

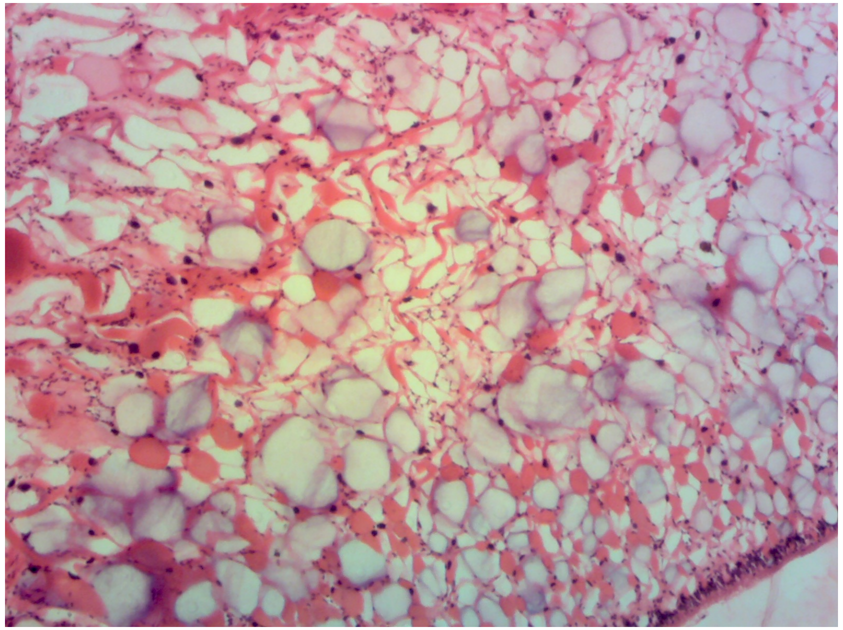

Section 1

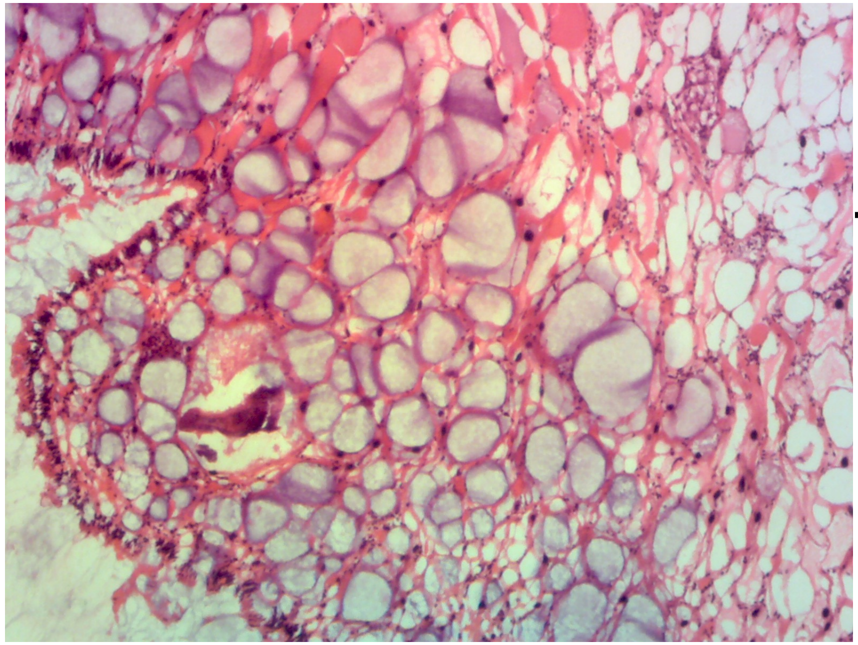

Section 2

Snail 2

Ovo-testis
Metalaxyl

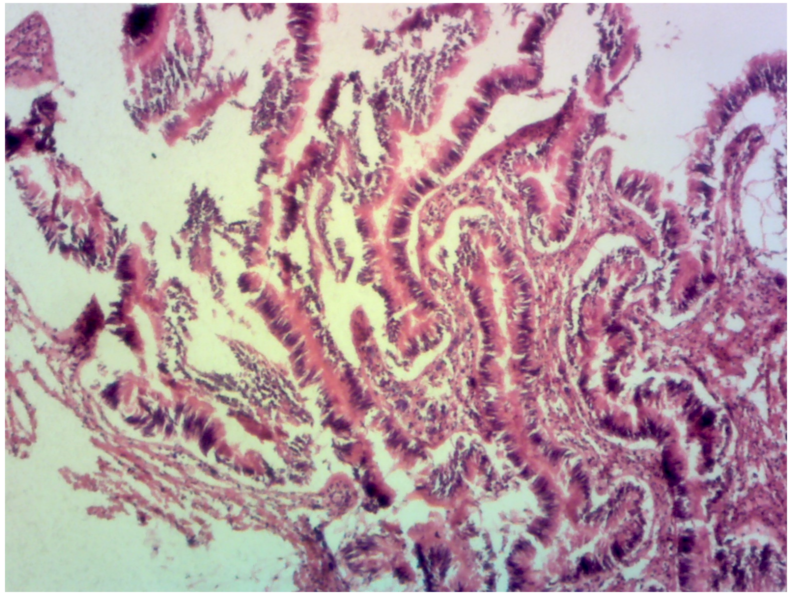

Section 1

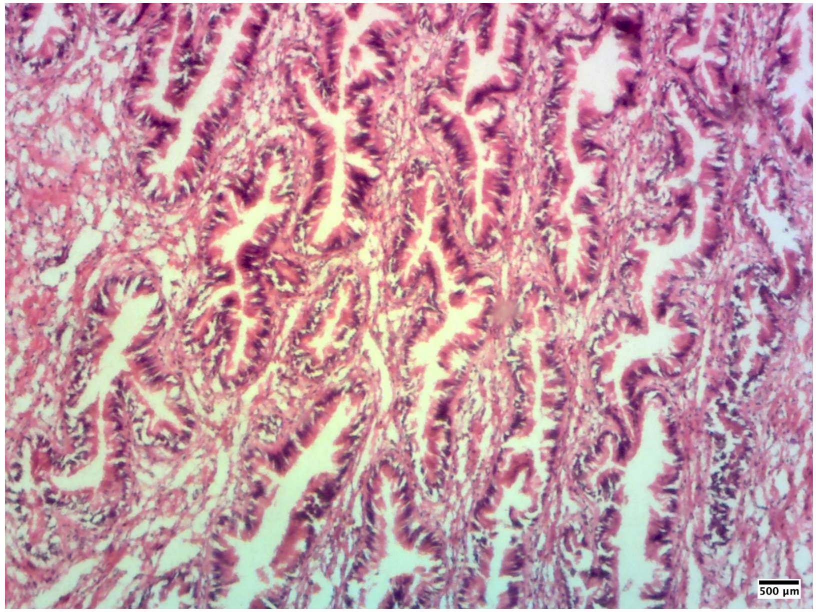

Section 2

Snail 1

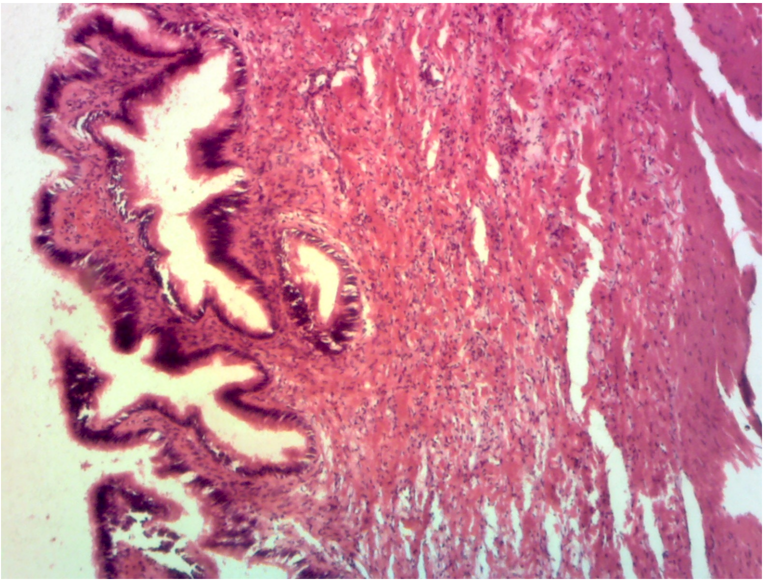

Section 1

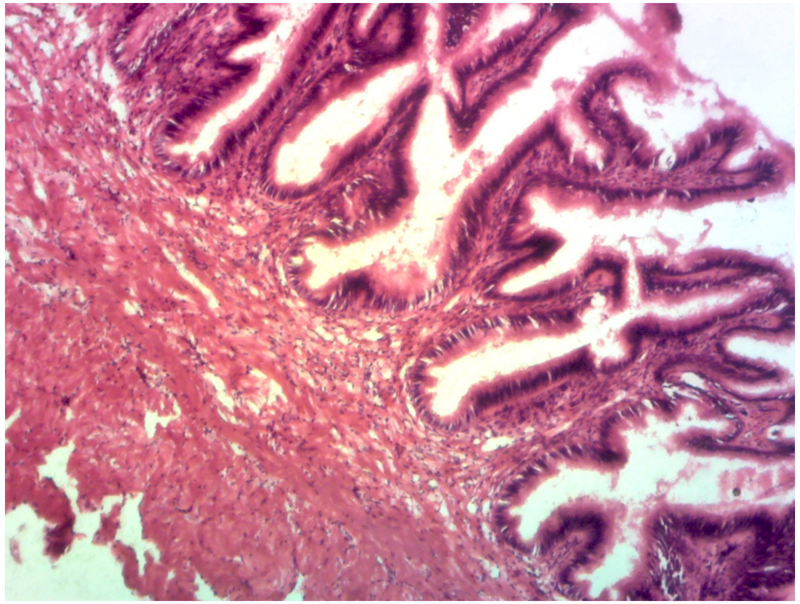

Section 2

Snail 2
